# Nomogram Based on Systemic Immune Inflammation Index and Prognostic Nutrition Index Predicts Recurrence of Hepatocellular Carcinoma After Surgery

**DOI:** 10.3389/fonc.2020.551668

**Published:** 2020-10-14

**Authors:** Junsheng Yang, Yongjin Bao, Weibo Chen, Yunfei Duan, Donglin Sun

**Affiliations:** Department of Hepatopancreatobiliary Surgery, The Third Affiliated Hospital of Soochow University, Changzhou, China

**Keywords:** hepatocellular carcinoma, recurrence, nomogram, SII, PNI

## Abstract

**Background:**

Surgery is a potential cure for hepatocellular carcinoma (HCC), but its postoperative recurrence rate is high, its prognosis is poor, and reliable predictive indicators are lacking. This study was conducted to develop a simple, practical, and effective predictive model.

**Materials and Methods:**

Preoperative clinical and postoperative pathological data on patients with HCC undergoing partial hepatectomies at the Third Affiliated Hospital of Soochow University from January 2010 to December 2015 were retrospectively analyzed, and a nomogram was constructed. The model performance was evaluated using C-indexes, receiver operating characteristic curves, and calibration curves. The results were verified from validation cohort data collected at the same center from January 2016 to January 2017 and compared with the traditional staging systems.

**Results:**

Three hundred three patients were enrolled in this study: 238 in the training cohort and 65 in the validation cohort. From the univariate and multivariate Cox regression analyses in the training cohort, six independent risk factors, i.e., age, alpha-fetoprotein (AFP), tumor size, satellite nodules, systemic immune inflammation index (SII), and prognostic nutritional index (PNI), were filtered and included in the nomogram. The C-index was 0.701 [95% confidence interval (CI): 0.654–0.748] in the training cohort and 0.705 (95% CI: 0.619–0.791) in the validation cohort. The areas under the curve for the 1- and 3-year recurrence-free survival were 0.706 and 0.716 in the training cohort and 0.686 and 0.743 in the validation cohort, respectively. The calibration curves showed good agreement. Compared with traditional American Joint Committee on Cancer 8th edition (AJCC8th) and Barcelona Clinic Liver Cancer (BCLC) staging systems, our nomogram showed better predictive ability.

**Conclusion:**

Our nomogram is simple, practical, and reliable. According to our nomogram, predicting the risk of recurrence and stratifying HCC patient management will yield the greatest survival benefit for patients.

## Introduction

Hepatocellular carcinoma (HCC) is the sixth most common cancer and the fourth leading cause of cancer-related death worldwide. Approximately 841,000 new cases and 782,000 deaths occur annually, presenting a public health burden (1). Surgery is a potential cure for HCC. However, the recurrence rate can reach 70% at 5 years postsurgery, and two-thirds of recurrences occur within 2 years (2). Traditional staging systems, such as the American Joint Committee on Cancer 8th edition (AJCC8th) and the Barcelona Clinic Liver Cancer (BCLC) staging systems, cannot satisfactorily predict postoperative prognosis (3). As a new clinical prognostic model, nomograms have been explored in several carcinomas (4–6). However, scholars have not reached a unified standard or consensus for a clinical prognosis model of an HCC nomogram. Therefore, a pragmatic and powerful standardized nomogram based on objective measures is needed to predict HCC prognoses.

Nomograms are based on multiple independent risk factors. Previous studies on tumor prognosis have been based primarily on demographic and clinicopathological data. Increasing attention is being given to the relationship between conventional serological indicators and tumor prognosis, including indicators of serum inflammation, serum nutrition, liver function, and coagulation function. Gan et al. (7) reported that fibrinogen and C-reactive protein scores were good prognostic indicators for postoperative patients with HCC. Ho et al. (8) showed that the albumin-bilirubin grade could predict HCC recurrence in patients after surgery.

Here, we constructed a practical and effective nomogram based on conventional prognostic indicators combined with multiple serological indicators to predict the postoperative recurrence of HCC.

## Materials and Methods

### Patients and Study Design

We retrospectively collected and analyzed preoperative clinical and postoperative pathological data for patients diagnosed with HCC who underwent partial hepatectomies at the Third Affiliated Hospital of Soochow University between January 2010 and January 2017. All data were collected in our hospital, and all serum indicators were obtained within 1 week before surgery. The inclusion criteria were (I) aged 20–85 years; (II) histopathologically proven HCC; and (III) initial diagnosis rather than recurrent tumors. Exclusion criteria were (I) acute tumor rupture with hemoperitoneum; (II) distant metastasis; (III) positive surgical margins; (IV) mixed cholangiocarcinoma; (V) other concomitant malignant diseases; (VI) perioperative death (A death occurred within 90 days after surgery) or death from other diseases during follow-up; (VII) incomplete data; and (VIII) lost to follow-up. Patients who were included from January 2010 to December 2015 were defined as the training cohort; patients who were included from January 2016 to January 2017 were defined as the validation cohort. The institutional review board of the Third Affiliated Hospital of Soochow University approved the study.

The model indices were calculated as follows. Body mass index was calculated by dividing the weight by the height squared (kg/m^2^). The neutrophil-to-lymphocyte ratio (NLR) was calculated by dividing the neutrophil count by the lymphocyte count. The lymphocyte-to-monocyte ratio was calculated by dividing the lymphocyte count by the monocyte count. The platelet-to-lymphocyte ratio (PLR) was calculated by dividing the platelet count by the lymphocyte count. The systemic immune inflammation index (SII) was calculated as platelet count × neutrophil count/lymphocyte count (10^9^/L). The systemic inflammatory response index (SIRI) was calculated as monocyte count × neutrophil count/lymphocyte count (10^9^/L). The prognostic nutritional index (PNI) was the sum of serum albumin (g/L) and 5 × lymphocyte count (10^9^/L).

### Follow-Up

All patients were advised to receive regular follow-up according to clinical guidelines after surgery. The outpatient review was conducted every 3 months for the first 2 postoperative years, then every 6 months thereafter if no recurrence or metastasis occurred. Serum AFP, liver function, routine blood, and abdominal ultrasound examinations were performed at each follow-up visit. If signs of recurrence were noted, further computed tomography (CT) examinations were conducted; otherwise, CT examinations were performed once every 6 months. The event endpoint was tumor recurrence. All tumor recurrences were diagnosed via CT images. Recurrence-free survival (RFS) was defined as the time interval between the date of surgery and the date that recurrence was diagnosed. The follow-up deadline was January 31, 2020.

### Statistical Analysis

Statistical analysis was performed using SPSS 19.0 (IBM Corp). Continuous variables were compared using the Mann–Whitney *U*-test; categorical variables were compared using chi-square tests. Continuous variables tested in the laboratory were divided into binary variables according to critical values. The cutoff values for age and model indices were obtained via receiver operating characteristic (ROC) curve analysis, and then these continuous variables were converted into binary variables according to cutoff values. Univariate and multivariate Cox regression analyses were used to identify independent risk factors for HCC recurrence in the training cohort. *P* < 0.05 was considered statistically significant.

A nomogram was constructed based on the multivariate Cox regression analysis results. The concordance index (C-index), ROC curves, and calibration curves were constructed to evaluate the model performance in the training cohort and verify it in the validation cohort. The risk score for recurrence was determined from the nomogram. The median score of the patients in the training cohort was defined as the cutoff value. Patients with values below the cutoff value were considered the low-risk group; patients with values above the cutoff value were considered the high-risk group. RFS was analyzed using the Kaplan–Meier method. The above steps were analyzed in R, version 3.6.0. The main R packages used included “rms,” “foreign,” “survival,” and “survivalROC.”

## Results

### Demographic and Clinical Characteristics

Three hundred three patients were enrolled in this study: 238 in the training cohort and 65 in the validation cohort. Table 1 lists the detailed demographics, serum indices, pathological characteristics, AJCC8th stage, and BCLC stage of the patients in the two cohorts.

**TABLE 1:** Demographic and clinical characteristics.

Variable	Training cohort (*n* = 238)	Validation cohort (*n* = 65)	*P*-value
Age (years)	59.1 ± 11.3	60.4 ± 11.6	0.429
Sex			0.425
Male	195	56	
Female	43	9	
BMI (kg/m^2^)	22.9 ± 3.0	23.5 ± 3.0	0.190
HBsAg			0.662
Positive	182	48	
Negative	56	17	
AFP (ng/ml)	323.2 ± 493.3	247.5 ± 438.6	0.263
ALT (U/L)	43.3 ± 35.9	37.4 ± 27.0	0.222
Bilirubin (μmol/L)	11.4 ± 5.0	14.1 ± 7.7	0.009*
Albumin (g/L)	38.3 ± 5.1	40.2 ± 4.3	0.008*
Leukocyte count (×10^9^/L)	5.5 ± 2.2	6.1 ± 2.5	0.068
Neutrophil count (×10^9^/L)	3.5 ± 1.8	4.1 ± 2.2	0.014*
Lymphocyte count (×10^9^/L)	1.4 ± 0.7	1.5 ± 0.6	0.751
Monocyte count (×10^9^/L)	0.4 ± 0.2	0.4 ± 0.1	0.164
Platelet count (×10^9^/L)	155.2 ± 74.8	163.0 ± 78.4	0.462
Tumor size (cm)	5.0 ± 3.0	5.6 ± 3.7	0.161
Tumor number			0.830
Single	225	61	
Multiple	13	4	
Differentiation			0.293
Low	80	18	
Middle	142	45	
High	16	2	
Vascular invasion			0.175
Yes	51	9	
No	187	56	
Satellite nodules			0.007*
Yes	27	16	
No	211	49	
AJCC8th			0.158
Stage I	175	52	
Stage II	49	8	
Stage III	9	5	
Stage IV	5	0	
BCLC			0.189
Stage 0	21	3	
Stage A	107	35	
Stage B	54	18	
Stage C	56	9	

*P-value < 0.05.

In the training cohort, the average age of the patients was (59.1 ± 11.3) years, 195 (81.9%) cases were male patients, most of them were infected with hepatitis B virus (182 cases, account for 76.5%), the average diameter of the tumors was (5.0 ± 3.0) cm, and 225 (94.5%) patients had a single tumor. Pathology showed that 93.3% of the tumors were low and middle differentiation, 21.4% of them had vascular invasion, and 11.3% of them had satellite nodules. According to the AJCC8th and BCLC stage, early-stage patients accounted for 94.1% (stage I and II) and 53.8% (stage 0 and A), respectively.

In the validation cohort, the average age of the patients was (60.4 ± 11.6) years, and 56 (86.2%) cases were male patients. Similarly, 73.8% of patients were infected with the hepatitis B virus. The average diameter of the tumors was (5.6 ± 3.7) cm, and 61 (93.8%) patients had a single tumor. Pathology showed that 96.9% of the tumors were low and middle differentiation, 13.8% of them had vascular invasion, and 24.6% of them had satellite nodules. According to the AJCC8th and BCLC stage, early-stage patients accounted for 92.3 and 58.5%, respectively.

Compared with the validation cohort, the serum bilirubin (11.4 ± 5.0 vs.14.1 ± 7.7 μmol/L, *P* = 0.009), albumin (38.3 ± 5.1 vs. 40.2 ± 4.3 g/L, *P* = 0.008), neutrophil counts (3.5 ± 1.8 vs. 4.1 ± 2.2 × 10^9^/L, *P* = 0.014), and tumor satellite nodules (11.3% vs. 24.6%, *P* = 0.007) in the training cohort were significantly lower. There was no significant difference in age, sex, BMI, hepatitis B virus infection, serum AFP, alanine aminotransferase (ALT), leukocyte count, lymphocyte count, monocyte count, platelet count, and the size, number, differentiation, vascular invasion, AJCC8th, and BCLC stage of tumors between the two groups.

### Tumor RFS in the Training and Validation Cohorts

**FIGURE 1:**
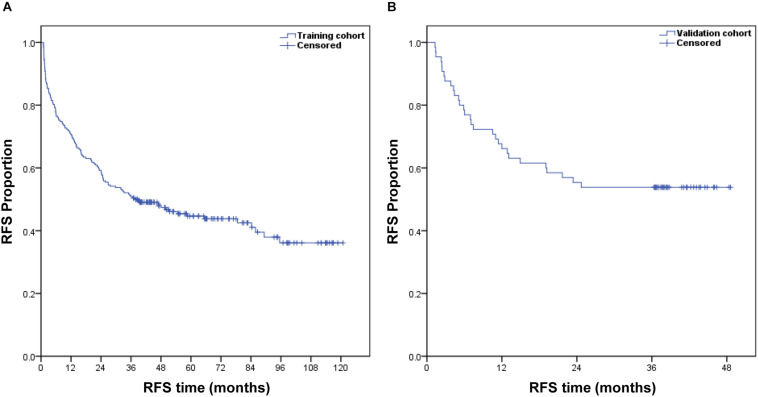
Kaplan–Meier estimate of the postoperative RFS of patients with HCC in the training cohort **(A)** and validation cohort **(B)**.

In the training cohort, the median follow-up time was 36.9 months (range, 0.9–120.8 months), and the postoperative 1- and 3-year RFS were 70.6 and 50.8%, respectively. In the validation cohort, the median follow-up time was 36.4 months (range, 1.3–48.6 months), and the postoperative 1- and 3-year RFS were 66.2 and 53.8%, respectively. Figure 1 shows the RFS curves for both groups.

### Independent Risk Factors in the Training Cohort

In the training cohort, the optimal cutoff values for age, NLR, MLR, PLR, SII, SIRI, PNI, and tumor size were determined to be 67 years and 2.71, 0.26, 78.24, 279.29, 0.93, 48.05, and 6.5 cm, respectively, as per the ROC curves. All variables were then divided into categorical variables and analyzed via univariate and multivariate Cox regression analysis. Table 2 shows the univariate and multivariate Cox regression analysis results. Univariate analyses revealed that age (*P* = 0.006), AFP (*P* = 0.002), SII (*P* = 0.03), PNI (*P* = 0.015), tumor size (*P* = 0.003), and satellite nodules (*P* = 0.003) were identified as significant prognostic factors for HCC recurrence. In multivariate analysis, age [hazard ratio (HR): 1.710; 95% confidence interval (CI): 1.083–2.702; *P* = 0.021], AFP (HR: 1.498; 95% CI: 1.041–2.156; *P* = 0.03), SII (HR: 1.456; 95% CI: 1.034–2.051; *P* = 0.031), PNI (HR: 1.503; 95% CI: 1.016–2.223; *P* = 0.041), tumor size (HR: 1.621; 95% CI: 1.109–2.369; *P* = 0.013), and satellite nodules (HR: 1.829; 95% CI: 1.140–2.933; *P* = 0.012) were identified as independent risk factors for HCC recurrence.

**TABLE 2:** Cox proportional hazards regression analyses of recurrence in the training cohort.

Variable	Cases	Univariate analysis		Multivariate analysis	
			
		HR (95% CI)	*P*-value	HR (95% CI)	*P*-value
Age (years)		1.878 (1.198–2.944)	0.006*	1.710 (1.083–2.702)	0.021*
≤67	183				
>67	55				
Gender		0.801 (0.507–1.266)	0.342		
Male	195				
Female	43				
BMI (kg/m^2^)		1.392 (0.961–2.018)	0.080		
<25	177				
≥25	61				
HBsAg		1.434 (0.927–2.217)	0.105		
Positive	182				
Negative	56				
AFP (ng/ml)		1.743 (1.219–2.492)	0.002*	1.498 (1.041–2.156)	0.030*
≤8	100				
>8	138				
ALT (U/L)		1.214 (0.862–1.710)	0.267		
≤40	145				
>40	93				
Bilirubin (μmol/L)		0.955 (0.390–2.334)	0.919		
≤20.5	228				
>20.5	10				
NLR		1.262 (0.880–1.812)	0.206		
≤2.71	147				
>2.71	91				
MLR		1.292 (0.913–1.828)	0.148		
≤0.26	105				
>0.26	133				
PLR		1.308 (0.915–1.869)	0.140		
≤78.24	70				
>78.24	168				
SII		1.455 (1.036–2.042)	0.030*	1.456 (1.034–2.051)	0.031*
≤279.29	102				
>279.29	136				
SIRI		1.360 (0.958–1.931)	0.085		
≤0.93	136				
>0.93	102				
PNI		1.615 (1.098–2.375)	0.015*	1.503 (1.016–2.223)	0.041*
≤48.05	157				
>48.05	81				
Tumor size (cm)		1.753 (1.205–2.550)	0.003*	1.621 (1.109–2.369)	0.013*
≤6.5	183				
>6.5	55				
Differentiation		1.993 (0.947–4.198)	0.069		
Low	80				
Middle	142				
High	16				
Vascular invasion		1.321 (0.891–1.958)	0.165		
Yes	51				
No	187				
Satellite nodules		2.046 (1.281–3.268)	0.003*	1.829 (1.140–2.933)	0.012*
Yes	27				
No	211				

*P-value < 0.05.

### Construction and Verification of the RFS Nomogram

**FIGURE 2:**
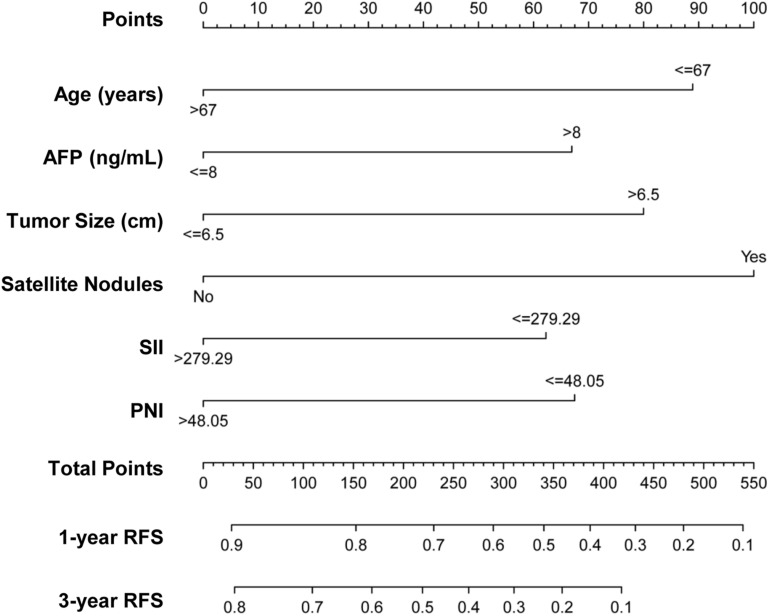
Nomogram predicting 1- and 3-year RFS probabilities of patients with HCC. Each risk factor was assigned a point according to the nomogram. The exact values of each factor are age (0, 89 points), AFP (0, 67 points), tumor size (0, 80 points), satellite nodules (0, 100 points), SII (0, 62 points), and PNI (0, 68 points). The total points were obtained by adding the points of all risk factors. Then, the 1- and 3-year RFS could be predicted based on the total points.

From the multivariate Cox regression analysis results, age, AFP, tumor size, satellite nodules, SII, and PNI were integrated and used to construct the nomogram (Figure 2), which showed that satellite nodules had the greatest impact on HCC recurrence, followed by age, tumor size, PNI, AFP, and SII. The total scores of these six prognostic factors can be used to determine the probabilities of 1- and 3-year RFS.

**FIGURE 3:**
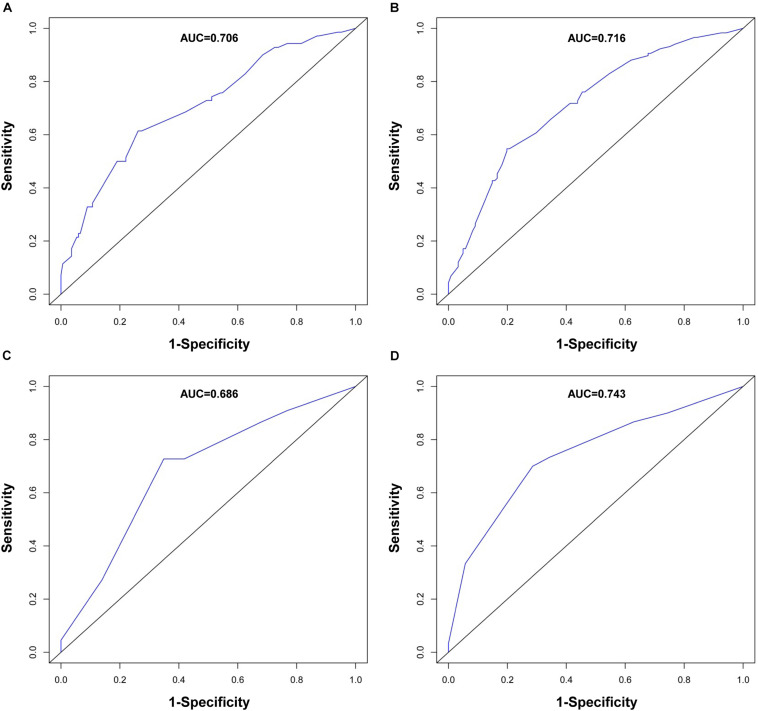
ROC curve evaluating predictive performance of the nomogram for 1-year RFS **(A)** and 3-year RFS **(B)** in the training cohort and 1-year RFS **(C)** and 3-year RFS **(D)** in the validation cohort.

**FIGURE 4:**
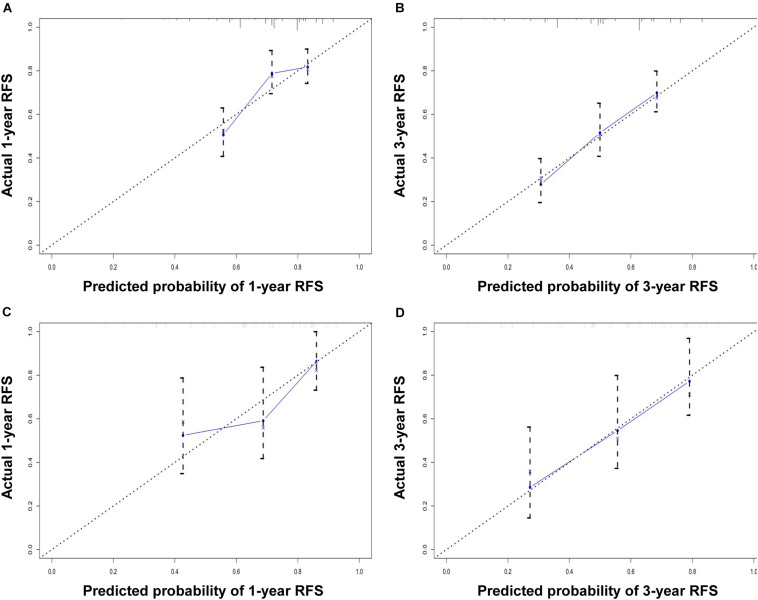
Calibration curve for predicting 1-year RFS **(A)** and 3-year RFS **(B)** in the training cohort and 1-year RFS **(C)** and 3-year RFS **(D)** in the validation cohort. The nomogram-predicted probability of RFS is plotted on the *x*-axis; the actual RFS is plotted on the *y*-axis.

The performance of the nomogram in the training cohort was evaluated using the C-index, ROC curves, and calibration curves. The C-index for RFS prediction was 0.701 (95% CI: 0.654–0.748). The areas under the ROC curve (AUCs) for the 1- and 3-year RFS were 0.706 and 0.716, respectively (Figures 3A,B). The calibration curves for the probability of 1- and 3-year RFS after surgery showed good probability consistencies between the nomogram prediction and actual observation (Figures 4A,B).

Using the same method, we verified the predictive ability of the nomogram in the validation cohort. The results showed that the C-index was 0.705 (95% CI: 0.619–0.791). The AUCs of the 1- and 3-year RFS were 0.686 and 0.743, respectively (Figures 3C,D). The calibration curves also had good prediction consistency (Figures 4C,D).

### Comparison of Predictive Ability of RFS in HCC Patients Between the Nomogram and Traditional Staging Systems

**FIGURE 5:**
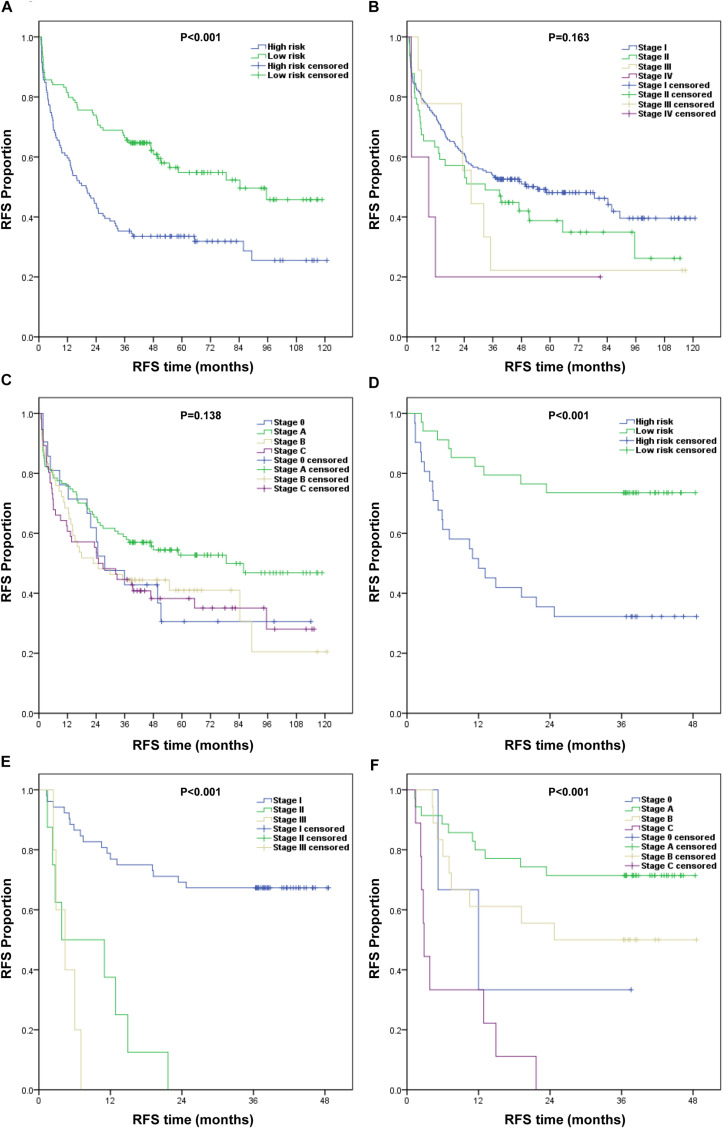
Kaplan–Meier survival curves of RFS for different models. **(A–C)** Training cohort: **(A)** nomogram, **(B)** AJCC8th staging system, and **(C)** BCLC staging system. **(D–F)** Validation cohort: **(D)** nomogram, **(E)** AJCC8th staging system, and **(F)** BCLC staging system.

First, we divided the patients into the low-risk and high-risk groups based on the median nomogram score (218.5 points) for the training cohort. The RFS was then analyzed using the Kaplan–Meier method. The RFS survival probability of the high-risk group was significantly lower than that of the low-risk group in both the training and validation cohorts (Figures 5A,D). RFS analysis was performed using the same method based on the AJCC8th and BCLC staging systems. RFS survival probability differed significantly between stages according to the AJCC8th and BCLC staging systems in the validation cohort (Figures 5E,F) but not in the training cohort (Figures 5B,C). Their predictive abilities were compared by calculating the C-index (Table 3). In the training cohort, the C-index of the nomogram (0.701; 95% CI: 0.654–0.748) was higher than that of the AJCC8th (0.533) and BCLC (0.548) staging systems. In the validation cohort, the nomogram performance (C-index: 0.705; 95% CI: 0.619–0.791) was also superior to the AJCC8th (0.672) and BCLC (0.684) staging systems. Therefore, our nomogram better predicted the RFS for HCC patients.

**TABLE 3:** Comparison of the predictive ability of models.

Model	Training cohort			Validation cohort		
		
	C-index	95% CI	*P*-value	C-index	95% CI	*P*-value
Nomogram	0.701	0.654–0.748	<0.001	0.705	0.619–0.791	<0.001
AJCC8th	0.533	0.494–0.572	0.163	0.672	0.599–0.745	<0.001
BCLC	0.548	0.499–0.597	0.138	0.684	0.588–0.780	<0.001

## Discussion

For resectable HCC, surgery is the best treatment option. However, high recurrence rates after hepatectomies greatly reduce patients’ long-term survival benefit. In the training cohort of this study, the 1- and 3-year HCC recurrence rates were 29.4 and 49.2%, respectively. Therefore, the risk of postoperative HCC recurrence must be predicted and stratified to allow early intervention for high-risk patients. At present, the internationally recognized and widely used systems include the AJCC, BCLC, Italian Liver Cancer Program, and Okuda staging systems. However, these traditional liver cancer staging systems have not achieved satisfactory results in predicting the prognosis (3, 9). The nomogram is a prognostic prediction map based on multiple independent risk factors. It has been researched in a variety of tumors, and several studies have reported that its prediction performance is better than that of traditional liver cancer staging systems (10, 11).

In this study, we established a nomogram based on SII and PNI. The nomogram showed good performance for predicting prognoses as per C-index, ROC curve, calibration curve, and internal cohort verification. The nomogram also exhibited better prognostic predictive ability compared with the traditional AJCC and BCLC staging systems.

Studies have shown that systemic immune inflammatory responses are significantly correlated with cancers (12, 13). Neutrophils in the peripheral blood or tumor microenvironment can produce angiogenic factors that stimulate tumor development and progression (14). Lymphocytes reflect the host immunity status and can inhibit tumor progression (15). Therefore, cancer-related inflammation is considered the seventh hallmark of cancer (16). Recent studies have mainly focused on the inflammation index converted from multiple serum inflammatory indicators, such as the NLR, MLR, PLR, SII, and SIRI, modified Glasgow prognostic score (mGPS), and C-reactive protein/albumin ratio (CAR). Nomogram clinical models based on inflammatory indicators such as NLR, PLR, hypersensitive C-reactive protein (hs-CRP), and FC-score composed of fibrinogen and C-reactive protein are reported to have good predictive prognostic performances for HCC (7, 17–19). In this study, several easily available inflammatory indicators were analyzed, including NLR, PLR, MLR, SIRI, and SII. Although the prognostic ability of CAR in HCC is reportedly better than that of other inflammatory indexes (10), C-reactive protein is not used in routine preoperative examinations in our center; thus, it was excluded from the study, as was the mGPS. Our results indicated that only the inflammatory index SII was an independent risk factor for postoperative HCC recurrence. Fu et al. (20) reported that preoperative SII was a powerful prognostic biomarker in HCC patients who undergo liver transplantation, and it was superior to PLR, NLR, and MLR for prediction of overall survival. Jomrich et al. (21) also showed that SII was superior to PLR and NLR for predicting overall survival in pancreatic ductal adenocarcinoma patients. Therefore, SII was a convenient, low-cost, and effective inflammatory indicator to predict the prognosis of tumors. Previous studies have only reported the use of SII in nomograms for gastric and tongue cancer (22, 23). Here, we report for the first time that nomograms based on SII can effectively predict postoperative HCC recurrence.

Relationships between nutritional status and cancer prognosis have gained attention from researchers over the last decade. Studies have shown that malnutrition often leads to poor tumor prognosis (24). In studying the HCC clinical nomogram model, albumin-based nutritional indexes, including the albumin-bilirubin grade and lactic dehydrogenase/albumin ratio, showed good prognostic predictive performance (25, 26). In this study, we explored the albumin-based PNI. The PNI was originally designed to assess the nutritional status of patients undergoing gastrointestinal surgery (27). Pinato et al. (28) found for the first time that the PNI independently predicted overall survival of HCC patients. Our results also indicated that low PNI (≤48.05) was an independent risk factor for postoperative HCC recurrence, and our PNI-based nomogram showed good prediction performance. Cancer cachexia is reportedly driven by a sustained inflammatory response (29). Therefore, we believe that inflammation and malnutrition jointly promote tumor progression; thus, our nomogram comprehensively includes both inflammation and nutrition indexes.

Our nomogram also included four common factors: age, serum AFP, tumor size, and satellite nodules; for these, we included demographic characteristics, serum indicators, and clinicopathological data to optimize the overall predictive ability.

At present, there was no uniform standard for predicting the prognosis of HCC. The AJCC and BCLC staging systems were the most common staging systems for liver cancer and were primarily meant to formulate patient’s treatment plans in oncology settings. In recent studies, Kee and Chun et al. (9, 30) reported that the AJCC staging system had a predictive ability for the prognosis of HCC. Grieco et al. (31) reported that the BCLC staging system gave a better prediction of prognosis in patients with HCC at a very early stage. Kim et al. (32) also found that the BCLC staging system was the best long-term prognostic model for treatment-naive HCC in a large scale Korean cohort. These reports indicated that AJCC and BCLC staging systems had predictive value for the prognosis of HCC. So, we compared the AJCC8th and BCLC staging systems with our nomogram model in our study. Combining the above six independent risk factors, our nomogram model showed a better predictive ability for postoperative recurrence of HCC than the AJCC8th and BCLC staging systems.

According to our nomogram, we can assess the risk of recurrence of HCC after surgery and conduct early individualized interventions for high-risk patients. We think that early interventions mainly include the following aspects: First, etiological interventions are critical, such as anti-hepatitis B virus, alcohol withdrawal, etc. Second, early postoperative transarterial chemoembolization (TACE) may play a role in preventing tumor recurrence, especially for patients with large and multiple lesions. Third, early postoperative immunoregulatory therapy may be a strategy, such as programmed cell death protein 1 (PD-1) inhibitor, thymosin, interferon, etc. Fourth, the detection of gene targets should be conducted after surgery, and targeted therapy may bring survival benefits to suitable patients. The early implementation of these methods may reduce the risk of tumor recurrence to a certain extent.

This study also had several limitations. First, this was a retrospective study. Second, our nomogram performance was verified with an internal cohort, without external or multicenter verification. Third, the number of patients enrolled in the model was relatively small. Therefore, further large-sample, multicenter, and prospective verification studies are needed. Besides, the exclusion of patients who were lost to follow-up was the main bias in this study.

In conclusion, our nomogram based on SII and PNI is simple, practical, and reliable. It has a good predictive ability for postoperative HCC recurrence. Our nomogram suggests that predicting the risk of recurrence and stratifying the management of HCC patients will yield the greatest survival benefit for patients.

## Data Availability Statement

The raw data supporting the conclusions of this article will be made available by the authors, without undue reservation.

## Ethics Statement

The studies involving human participants were reviewed and approved by the institutional review board of the Third Affiliated Hospital of Soochow University. Written informed consent for participation was not required for this study in accordance with the national legislation and the institutional requirements.

## Author Contributions

DS conceived and designed the study. JY collected the data and wrote the manuscript. YB analyzed the data. YD and WC modified the manuscript. All co-authors have reviewed and approved this version of the manuscript. All authors contributed to the article and approved the submitted version.

## Conflict of Interest

The authors declare that the research was conducted in the absence of any commercial or financial relationships that could be construed as a potential conflict of interest.
